# Beyond Low Prevalence: Exploring Antibiotic Resistance and Virulence Profiles in Sri Lankan Helicobacter pylori with Comparative Genomics

**DOI:** 10.3390/microorganisms13020420

**Published:** 2025-02-14

**Authors:** Kartika Afrida Fauzia, Jeewantha Rathnayake, Dalla Doohan, Meegahalande Durage Lamawansa, Ricky Indra Alfaray, Saruuljavkhlan Batsaikhan, Bui Hoang Phuc, Langgeng Agung Waskito, Vo Phuoc Tuan, Evariste Tshibangu Kabamba, Shamshul Ansari, Takashi Matsumoto, Junko Akada, Takeshi Matsuhisa, Yoshio Yamaoka

**Affiliations:** 1 Department of Environmental and Preventive Medicine, Oita University Faculty of Medicine, Yufu 879-5593, Japan; kartikafauzia@gmail.com (K.A.F.); doctordoohan@gmail.com (D.D.); rickyindraalfaray@gmail.com (R.I.A.); saruuljavkhlan@yahoo.com (S.B.); langgengaw@gmail.com (L.A.W.); vophuoctuandr@gmail.com (V.P.T.); evaristetshibangu@gmail.com (E.T.K.); shamshulansari483@yahoo.com (S.A.); tmatsumoto9@oita-u.ac.jp (T.M.); akadajk@oita-u.ac.jp (J.A.); 2 Research Center for Preclinical and Clinical Medicine, National Research and Innovation Agency, Bogor 16915, Indonesia; 3 *Helicobacter pylori* and Microbiota Study Group, Institute of Tropical Disease, Universitas Airlangga, Surabaya 60115, Indonesia; 4 Department of Surgery, University of Peradeniya & Teaching Hospital Peradeniya, Kandy 2017, Sri Lanka; jeewanrath@gmail.com (J.R.); mdyasas@yahoo.co.uk (M.D.L.); 5 Department of Anatomy, Histology and Pharmacology, Faculty of Medicine, Universitas Airlangga, Surabaya 60115, Indonesia; 6 Faculty of Applied Technology, School of Technology, Van Lang University, Ho Chi Minh 700000, Vietnam; phuc.bui@vlu.edu.vn; 7 Department of Endoscopy, Cho Ray Hospital, Ho Chi Minh 749000, Vietnam; 8 Research Center for Infectious Sciences, Department of Parasitology, Graduate School of Medicine, Osaka City University, Osaka 585-8585, Japan; 9 Health Science Division, Higher Colleges of Technology, Abu Dhabi Campus, Abu Dhabi 25026, United Arab Emirates; 10 Department of Gastroenterology, Nippon Medical School Tama Nagayama Hospital, Tama 206-8512, Japan; matuhisa@m8.dion.ne.jp; 11 Department of Internal Medicine, Faculty of Medicine, Universitas Airlangga, Surabaya 60115, Indonesia; 12 Department of Medicine, Gastroenterology and Hepatology Section, Baylor College of Medicine, Houston, TX 77030, USA; 13 The Research Center for GLOBAL and LOCAL Infectious Diseases (RCGLID), Oita University, Yufu 879-5593, Japan

**Keywords:** *Helicobacter pylori*, antibiotic resistance, virulence factors, comparative genomics

## Abstract

*Helicobacter pylori* infects at least half the population worldwide, and its highly diverse genomic content correlates with its geographic distribution because of its prolonged relationship with humans. The extremely low infection prevalence alongside low inflammation severity observed in some countries might be caused by strains with low virulence potential. Therefore, this study aimed to investigate whole-genome analysis datasets of Sri Lankan *H. pylori* strains. *H. pylori* strains were isolated from biopsy specimens and underwent whole-genome sequencing to investigate their antibiotic resistance and virulence potential. The prevalence of *H. pylori* infection in Sri Lanka is extremely low (1.7% in a previous study), and only six *H. pylori* strains were successfully isolated from bacterial culture. Antibiotic resistance analysis showed a high prevalence of metronidazole resistance (83.3%, five out of six strains), and investigation of the related genes showed truncation of the *rdxA* and *frxA* genes and single-nucleotide polymorphisms in the *rdxA*, *frxA*, *ribF*, *omp11*, and *fur* genes. Most virulence genes of the 144 assessed were present, except for the *cag* pathogenicity island (*cag*PAI) (absent in four out of six strains), *babA/B/C*, and *tlpB* genes. An incomplete type 4 secretion system (*tfs*) was found in three strains. A pan-genome analysis with non-Sri Lankan *H. pylori* strains showed that the *htpX* gene was found only in Sri Lankan strains (*p*-corrected = 0.0008). A phylogenetic analysis showed that the Sri Lankan strains clustered with strains from hpAsia2 and hpEurope. This comparative genomic study shows that *H. pylori* strains with low virulence potential are present in countries with a low prevalence of infection and disease severity, indicating a strain-type geographical pattern. The tailored guidelines for screening and treatment strategy for each region are necessary to obtain effective and efficient eradication.

## 1. Introduction

Around 50% of the world’s human population is infected with *Helicobacter pylori*, a carcinogenic pathogen [1]. *H. pylori* infection represents a serious public health problem because it has a causal relationship with gastric cancer and gastric mucosal-associated lymphoid tissue (MALT) lymphoma [2]. Over years of persistent *H. pylori* infection in humans, co-evolution has led to adaptation to the unique environment of the human gastric lumen, resulting in extreme genomic diversity [3]. The diversity of *H. pylori* is also reflected in its causal effect on humans. Infection prevalence varies between countries, and even between regions within a country. The probability of developing disease among *H. pylori-*infected patients tends to be widely variable, consistent with the geographic pattern of *H. pylori* distribution [4,5,6]. *H. pylori* infection in the South Asia region is known to be prevalent but the gastric cancer rate was low compared to the rate reported in the East Asia region [6].

The genetic variation observed in *H. pylori* strains is attributed to their long-term interaction with the host. Factors such as the ethnicity, residence, and antibiotic consumption patterns of the host are thought to influence the adaptation of *H. pylori*, as reflected in its genetic diversity [7]. This genetic variation can now be assessed more comprehensively by evaluating whole-genome sequences, as technologies become more advanced and more feasible [8,9]. Current advances in next-generation sequencing technologies have enabled researchers to assemble and screen multiple genes, evaluate the synteny, and perform comparative genomic analyses [10]. This approach is ideal for a comprehensive description of the virulence properties of *H. pylori*, especially in countries where *H. pylori* has been newly reported. Thus, this technology is beneficial for the development of precision *H. pylori* therapy. The most valuable genetic variation data are related to antibiotic resistance and virulence factors [9]. This information can be used to mitigate the risk of infection and assist in the development of more precise treatment guidelines tailored to specific regions or countries.

Virulence genes are required to colonize, adapt, persist, and propagate inside the host, and genetic variations contribute to the induction of inflammation [11]. One of the crucial virulence factors is cytotoxic-associated gene A (CagA), an oncogenic protein that is injected through the type 4 secretion system (Tfs) and enables *H. pylori* to induce a dysmorphic phenotype internally in gastric epithelial cells [12]. CagA has variable EPIYA motifs with varying affinity to SHP2; these follow a geographic pattern that partially explains the different rates of carcinogenesis between different geographical regions [12]. Vacuolating cytotoxin A (VacA) is a pore-forming toxin that causes vacuolation in gastric epithelial cells [8]. VacA enhances the capability of *H. pylori* to colonize the stomach and contributes to the pathogenesis of *H. pylori*-induced diseases [9]. The urease-family genes, flagella apparatus-encoding genes, and the chemotaxis gene are involved in early colonization and persistence in the acidic environment of the human stomach [13]. Genomic analysis has already screened *H. pylori* for well-known virulence genes, such as *cagA*, *vacA*, and several outer membrane proteins, including genes encoding the blood-group antigen-binding adhesin BabA [14]. There is also evidence supporting the association of insertion sequences, phage elements, and plasticity regions (such as the *cag* pathogenicity island, *cag*PAI) with disease severity [15,16]. Several genes related to antibiotic resistance have been extensively studied and can be used as biomarkers to detect antibiotic resistance. For example, 23S rRNA and *gyrA* are markers for clarithromycin (CLA) and levofloxacin (LEV) resistance, respectively [17].

A recent investigation on the seroprevalence of *H. pylori* infection in Sri Lanka, a country in South Asia that is separated from the Asian continent, revealed an extremely low *H. pylori* infection rate [18]. Furthermore, an endoscopy study found no cases of duodenal ulcers or gastric cancer [18]. The resistance profiles possessed by the strains found in this low-gastric-cancer environment are also crucial. A genomic dataset of Sri Lankan strains, together with histological evaluation of biopsy specimens for disease severity, could be an interesting model to understand the genomic profile of *H. pylori* from regions with a low prevalence of severe disease. Moreover, understanding the virulence genes possessed by these strains is equally important. Therefore, this study was conducted to elucidate the antibiotic resistance and virulence potential of Sri Lankan *H. pylori* strains, based on a whole-genome approach in strains associated with low infection prevalence and disease severity.

## 2. Materials and Methods

### 2.1. Sample Collection and Bacterial Strains

The isolates evaluated in this study were based on a clinical survey in Sri Lanka from 2017 [18]. In the survey, 353 patients with dyspeptic symptoms who visited Paradeniya Hospital, Sri Lanka, were recruited for endoscopy consecutively. Biopsy specimens were collected from the antrum, body, and angulus following the modified version of the Updated Sydney System to evaluate the *H. pylori* [19,20,21]. Patients with no previous history of *H. pylori* eradication and gastric resection were included and provided written informed consent for the study [18]. However, the prevalence of *H. pylori* infection was very low; only six strains could be isolated from all subjects. The strains were isolated from patients from different ethnicities as follows: Muslim, SLK36; Tamil, SLK231; and Sinhalese, strains SLK40, SLK91, SLK231, and SLK237.

All the study protocols of this study were approved by the ethical committee of Paradeniya Hospital, Sri Lanka, and the Faculty of Medicine, Oita University, Yufu, Japan, in accordance with the Declaration of Helsinki. The biopsy specimens for histology analysis were fixed in 10% formalin and embedded in liquid paraffin to form a block. Hematoxylin–eosin and Giemsa staining were performed to determine the gastric inflammation level according to the Updated Sydney System [20,21]. Gastritis was evaluated in the biopsy specimens collected from the antrum, body, and angulus. Neutrophil infiltration, monocyte infiltration, atrophy, and intestinal metaplasia were evaluated in each location. Each of these parameters was graded as follows: 0, none; 1, mild; 2, moderate; 3, severe. The Operative Link on Gastritis Assessment (OLGA) score was calculated by the sum of the atrophy grade/score in the antrum, body, and angulus.

For *H. pylori* isolation, a gastric biopsy from the antrum was taken and stored in *Brucella* broth medium (Becton, Dickinson and Company, Sparks, NV, USA) supplemented with 10% glycerol. After transportation to the laboratory, the biopsy specimens were homogenized and inoculated on *H. pylori*-selective culture plates (Eiken Chemical Co., Ltd., Tokyo, Japan). Strains growing with purple-colored colonies were collected and used for whole-genome sequencing and other in vitro analyses.

### 2.2. Next-Generation Sequencing and H. pylori Virulence Factors

DNA was extracted from a bacterial suspension of strains isolated from different patients using a Qiagen DNeasy Blood and Tissue Kit following the manufacturer’s instructions. Whoe-genome sequencing was performed using a high-throughput next-generation sequencer (Illumina MiSeq, Illumina, San Diego, CA, USA). The Illumina MiSeq provided paired-end short-read results that were used for further analyses. The short reads were trimmed using Trimmomatic version 0.39 and assembled using Unicycler v3.13.1 [22]. The sequences were then annotated by DFAST [23] and the open reading frames were used to determine the virulence genes. The quality of the assembly was assessed by Quast [24] and DFAST, with the minimum total length being 1.5 million bp, completeness 95%, and 0% contamination [23]. The presence of virulence genes was assessed by abricate (https://github.com/tseemann/abricate, accessed on 18 November 2023) using databases from the Virulence Factors Database (VFDB) [25] and Victors [26], with a minimum identity of 70% and minimum coverage of 50%. The *cagA*, *vacA*, and *oipA* genes were extracted by the BLASTN algorithm. The sequences were aligned and visualized by MEGA version 7.0 [27] to evaluate the genotype and “on” or “off” status. Plasmids, phages, and insertion sequences (ISs) were predicted by plasmidfinder 2.1.1, phaster v2.2, and ISFinder, the 2022 version, respectively [28,29,30]. The type 4 secretion system was detected by BLAST version 2.15 and visualized by CGview version 2.03 [31].

### 2.3. Comparative Genomic Analysis

The pan-genome analysis used GFF files from DFAST as input files for Roary [32], with a split-paralog mode and a minimum identity of 70%, to evaluate the core genomes and strain-specific genes. The results were visualized on phandango [33]. To assess the phylogenetic relatedness of *cagA* and *vacA*, a neighbor-joining tree using the Kimura-2 parameter was constructed using the *cagA* and *vacA* sequences obtained from the reference sequences, for which the genome populations had previously been determined [34]. We used complete genome sequences of *H. pylori* strains collected from various parts of the world as the references. The names and accession numbers of these reference genome sequences are as follows: 26695, NC_000915.1 (https://www.ncbi.nlm.nih.gov/nuccore/NC_000915.1); J99, NC_000921.1 (https://www.ncbi.nlm.nih.gov/nuccore/NC_000921.1); HPAG1, NC_008086.1 (https://www.ncbi.nlm.nih.gov/nuccore/NC_008086.1); Shi470, NC_010698.2 (https://www.ncbi.nlm.nih.gov/nuccore/NC_010698.2); G27, NC_011333.1 (https://www.ncbi.nlm.nih.gov/nuccore/NC_011333.1); P12, NC_011498.1 (https://www.ncbi.nlm.nih.gov/nuccore/NC_011498.1); F57, DDBJ: AP011945 (https://www.ncbi.nlm.nih.gov/nuccore/AP011945); F32, DDBJ: AP011943 (https://www.ncbi.nlm.nih.gov/nuccore/AP011943); F30, DDBJ: AP011941 (https://www.ncbi.nlm.nih.gov/nuccore/AP011941); F16, DDBJ: AP011940 (https://www.ncbi.nlm.nih.gov/nuccore/AP011940); B38, NC_012973.1 (https://www.ncbi.nlm.nih.gov/nuccore/NC_012973.1); 51, CP000012.1 (https://www.ncbi.nlm.nih.gov/nuccore/CP000012.1); v225d, CP001582.1 (https://www.ncbi.nlm.nih.gov/nuccore/CP001582.1); B8, NC_014256.1 (https://www.ncbi.nlm.nih.gov/nuccore/NC_014256.1); SJM180, NC_014560.1 (https://www.ncbi.nlm.nih.gov/nuccore/NC_014560.1); PeCan4, NC_014555.1 (https://www.ncbi.nlm.nih.gov/nuccore/NC_014555.1); Cuz20, CP002076.1 (https://www.ncbi.nlm.nih.gov/nuccore/CP002076.1); Sat464, CP002071.1 (https://www.ncbi.nlm.nih.gov/nuccore/CP002071.1); OK113, DDBJ: AP012600 (https://www.ncbi.nlm.nih.gov/nuccore/AP012600); OK310, DDBJ: AP012601 (https://www.ncbi.nlm.nih.gov/nuccore/AP012601); 35A, CP002096.1 (https://www.ncbi.nlm.nih.gov/nuccore/CP002096.1); 83, CP002605.1 (https://www.ncbi.nlm.nih.gov/nuccore/CP002605.1); Gambia94/24, CP002332.1 (https://www.ncbi.nlm.nih.gov/nuccore/CP002332.1); India7, CP002331.1 (https://www.ncbi.nlm.nih.gov/nuccore/CP002331.1); Lithuania75, CP002334.1 (https://www.ncbi.nlm.nih.gov/nuccore/CP002334.1); Puno120, CP002980.1 (https://www.ncbi.nlm.nih.gov/nuccore/CP002980.1); Puno135, CP002982.1 (https://www.ncbi.nlm.nih.gov/nuccore/CP002982.1); Santal49, CP002983.1 (https://www.ncbi.nlm.nih.gov/nuccore/CP002983.1); and NAB47, AJFA00000, (all URL were accessed on 5 January 2023).

### 2.4. Adherence Test and Hummingbird Phenotypes

An adherence and invasion test was performed based on a previous study with slight modifications [35,36]. Each strain was cultured on *Brucella* agar supplemented with 7% horse blood. A standardized bacterial concentration with an optical density (OD) of 0.07 of Sri Lankan strains together with reference strain 26,695 were inoculated into a gastric epithelial (AGS) cell line with a confluence of more than 80% in 12-well plates to produce a multiplicity of infection (MOI) of 50. This co-culture was incubated for 4 h before the medium was replaced with RPMI 1640 Medium (Gibco, Thermo Fisher Scientific, Waltham, MA, USA) supplemented with 10% fetal bovine serum (FBS) with 100 mg/μL gentamycin for 1 h. The cells were washed with phosphate-buffered saline (PBS) and added to RPMI supplemented with 10% FBS and 10 mg/μL gentamycin for incubation for 24 h. The cells were then incubated with 0.1% saponin (Sigma Aldrich, Darmstadt, Germany) to break the attachment between the cells and the plate. The supernatant was diluted 10, 100, and 1000 times with PBS and inoculated onto *Brucella* agar (Becton Dickinson, Carlsbad, CA, USA) supplemented with 7.0% FBS. The number of colonies grown was evaluated after 5 days. For hummingbird phenotype analysis, strains were co-cultured in the AGS cell line for 6 h. The hummingbird phenotype was defined as AGS cell elongation with a height–width proportion of around 1:5, evaluated at 100-times magnification [37].

### 2.5. Antibiotic Susceptibility Test

An antibiotic susceptibility test against five major *H. pylori* antibiotics—amoxicillin (AMX), clarithromycin (CLA), metronidazole (MNZ), levofloxacin (LEV), and tetracycline (TCN)—was performed using an E-test (Biomerieux, Marcy I’Etoille, France) [18]. Briefly, an *H. pylori* suspension from fresh culture was adjusted to be equivalent to 3.0 McFarland and then inoculated onto Mueller–Hinton II agar (Becton Dickinson, USA). An E-test strip was placed onto the middle of each agar plate and then incubated at 37 °C under microaerophilic conditions (10% O_2_, 5% CO_2_, and 85% N_2_). The minimum inhibitory concentration (MIC) of *H. pylori* strains towards each antibiotic was determined after 72 h. The determination of a strain as resistant or susceptible was made following the European Committee on Antimicrobial Susceptibility Testing (EUCAST) criteria of MIC > 0.125 mg/L for AMX, MIC > 0.5 mg/L for CAM, MIC > 8 mg/L for MNZ, MIC > 1 mg/L for LEV, and MIC > 1 mg/L for TCN [38].

## 3. Results

### 3.1. H. pylori Infection and Gastric Inflammation Severity

Of the six strains cultured, four strains were isolated from male patients and two from female patients, with a median age of 43.5 years (range: 33–62 years). All subjects infected with *H. pylori* were diagnosed with superficial gastritis. The scores for gastric inflammation according to the Updated Sydney System are shown in Supplementary Figure S1. Intestinal metaplasia was zero in all subjects. The patient infected with strain SLK231 had a prominent inflammation score and showed very high (severe) monocyte infiltration and high atrophy, especially in the gastric body. The highest OLGA score was observed in the patient infected with strain SLK231 (score 4), followed by those infected with strains SLK40 (score 3) and SLK237 (score 2). None of the strains showed any signs of intestinal metaplasia.

### 3.2. General Genomic Features of Sri Lankan Strains

**Figure 1:**
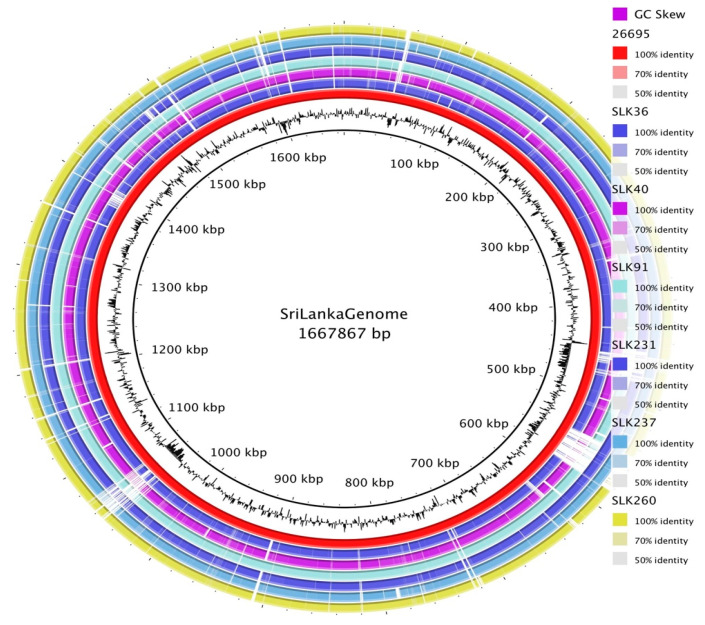
Whoe-genome sequences of six Sri Lankan strains. The annotated genomes of the Sri Lankan strains (n = 6) were compiled and a pan-genome was built.

All *H. pylori* strains isolated from Sri Lanka were sequenced by Illumina MiSeq. The sizes of the draft genomes ranged from 1,534,541 bp to 1,625,743 bp, with a GC percentage of 38.8–39.2%. Although the number of contigs ranged from 29 to 48, the longest N50 of the draft genomes was 137,496 bp (Supplementary Table S1). The average coding ratio was 89.7%, similar to the reference strain 26,695, which had a value of 90.5%. Each genome had 36 tRNA and two rRNA genes. Clustered regularly interspaced short palindromic repeats (CRISPRs) were also detected in all strains except SLK40 and SLK260. To examine the conserved genomic regions, the genomes were aligned to the genome of strain 26,695 (Figure 1). A total of 104 paralogous genes belonging to strain 26,695 were absent in all Sri Lankan strains. The alignments also showed that this missing region possessed a high GC skewness, indicating that it may be a region of plasticity.

Annotation showed that the genomes of the Sri Lankan strains had an average number of coding sequences of around 1470 genes, and that 1213 of those genes were possessed by all strains and thus defined as core genes. The total number of unique genes specific to each strain was 374 (Supplementary Figure S2).

### 3.3. Pan-Genome Analysis of Sri Lankan Strains

**Figure 2:**
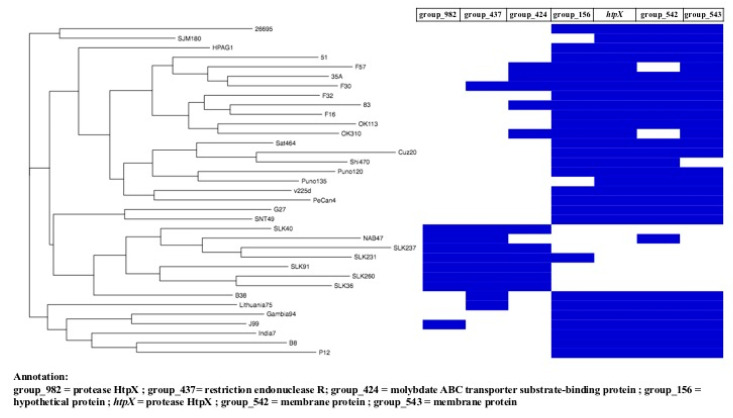
Gene presence matrix in Sri Lankan strains compared with reference genomes by the split-paralog mode. The left side of the tree is constructed using accessory genes from Roary, and the presence/absence matrix on the right side shows the genes with a specific link to Sri Lanka.

To further investigate the characteristics of the genes that were specific to only the Sri Lankan strains, a pan-genome analysis was performed in comparison with representative genomes belonging to the main *H. pylori* population (hpAfrica1, hpAsia2, hpEurope, and hpEastAsia) [34]. The pan-genomes were obtained by Roary with a minimum identity of 70% (Figure 2). The tree constructed from the binary accessory genes showed that the genomes of all the Sri Lankan strains were concentrated in a cluster that also included the Indian strains NAB47 and SNT49, from hpEurope and hpAsia2, respectively (Figure 2) [34,39,40]. The SLK231, SLK237, and SLK40 strains clustered with hpAsia2, while SLK36 and SLK91 clustered with hpEurope.

Data on the presence and absence of genes were used to determine the genes that are specifically present only in Sri Lankan strains. In the split-paralog mode, screening using Scoary found 122 genes with a naive *p*-value < 0.05. Benjamini–Hochberg correction (*p*-corrected) showed that six genes had a statistically significant association with the Sri Lankan strains. Genes annotated as protease HtpX (*group_982*, *p*-corrected = 0.008) and restriction endonuclease R (*group_437*, *p*-corrected = 0.046) were present in all Sri Lankan strains but absent in 93.1% (27/29) and 86.2% (25/29), respectively, of the other strains (Figure 2). Interestingly, two variant types of *htpX* were found, *group_982* and *original htpX.* The *original htpX* gene was present in 96.6% (28/29) of the non-Sri Lankan strains and absent in all Sri Lankan strains (*p*-corrected = 0.008). These results suggest an adaptation at the gene level to hosts in a particular geographic location.

In the non-split-paralog mode, an outer membrane protein, Omp22 (HP0923), was absent in all Sri Lankan strains but present in the others (false discovery rate (FDR) = 0.0006). These results reflect a wide range of genomic diversity that characterizes this specific geographic location.

### 3.4. Presence of H. pylori Virulence Factors and Genotypes

Comparative analysis of virulence factors using databases revealed the genes with various virulence functions, summarized in Supplementary Figure S3. We examined 144 virulence genes listed in the VFDB and Victor databases. We included as virulence factors not only genes related to toxicity to the host, but also genes supporting the processes of colonization and survival in chronic infection. Most adherence genes were present, except for *babA*, which was absent in four of the six strains. In our previous study, the presence of *babA* in *locus A* was significantly associated with an increased severity of inflammation [41]. Hence, further analysis of the locus-wise presence of the *bab* genes was performed (Supplementary Figure S4). The *babA* genes in SLK40 and SLK231 were in *locus A.* The only *babC* gene in strain SLK260 was in *locus A* after the *hypD* gene (a marker gene for *locus A*), whereas four strains (SLK40, SLK231, SLK237, and SLK260) harbored the *babB* gene only in *locus B*.

Genome annotation and confirmation by BLASTN revealed that the *cagA* gene was only present in two strains: SLK231 and SLK237 (two out of six; 33.3%). The *cag*PAI-negative Sri Lankan strains clustered with hpEurope (three strains) and hpAsia2 (one strain). Meanwhile, *vacA* was present in all strains. *vacA* consists of a signal region (s), a p33 domain (i, c, and d), and a p55 (m) domain. Both the *cagA*-positive Sri Lankan strains possessed the s1a i1 d1 m1 c1 *vacA* genotype, while all *cagA*-negative strains possessed the s2 i2 d2 m2 c2 genotype. We constructed a phylogenetic tree of the complete *vacA* gene to understand its variation compared with reference genomes worldwide (Supplementary Figure S5). The *vacA* s2 m2 genotype formed a cluster with *vacA* genes from India (SNT49, India7), France (B8, B38), and Peru (Cuz20), while the *vacA* s1 m1 genotype was found in a large cluster with strains from Japan, separated as a distinct branch.

*H. pylori* virulence factors, such as *oipA*, *babA*, *babB*, and *iceA*, were also genotyped. *oipA*, the gene encoding the outer membrane inflammatory protein, was present in all the isolated strains; however, a variable number of CT repeats was observed (Table 1). Among the six strains, four showed *oipA* with an ‘on’ status, while the other two had an *oipA* ‘off’ status.

### 3.5. CagA Genotype Specific to Strains from South Asia

**Figure 3:**
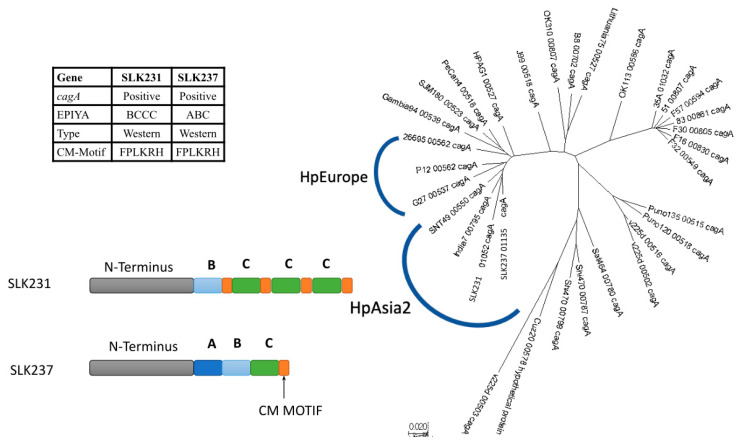
The *cagA* gene from Sri Lanka shows a BCCC genotype. Phylogenetic tree showing SLK231 and SLK237 clustered with the India7 *cagA* sequence.

Sequence analysis of the two *cagA*-positive strains, focusing on the EPIYA region, showed the typical western-type CagA with an ABC-type EPIYA motif in one strain, and a BCCC-type EPIYA motif in the other (Figure 3). We identified that the sequence following the first EPIYA motif matched the B-specific sequence, and the other EPIYA motifs matched the C-specific sequence (Supplementary Figure S6). Both strains possessed the typical CagA-multimerization (CM) sequence (FPLKRHDKVVNDL) of western-type CagA.

To further understand the phylogeny of *cagA*, a neighbor-joining tree was constructed from the full *cagA* sequences of strains SLK231 and SLK237 together with the full *cagA* sequences extracted from the reference genomes used in the pan-genome construction (Figure 3) and *vacA* phylogeny analysis in the previous section. The *cagA* sequences of strains SLK231 and SLK237 clustered together with the Indian strains India7 and SNT49, belonging to hpAsia2 (Figure 3). Even though this phylogenetic tree was constructed using only the *cagA* sequences, it was concordant with the genomic population and geographical region.

To assess the ability of the strains to damage host cells, we investigated the hummingbird phenotype of AGS cells after six hours of co-culture with different CagA motifs. The results showed that strain SLK231 resulted in a greater number of AGS cells with the hummingbird phenotype and dead cells compared with strains SLK237 and SLK36. Moreover, strain SLK231 caused a greater number of cells to show the hummingbird phenotype than strain SLK237 (Supplementary Figure S7).

As most of the strains from Sri Lanka were *cag*PAI-negative, we further evaluated the synteny of *cag*PAI. *cagA* and *cag*PAI are proposed to be insertion sequences to the *H. pylori* genome between the *dapB* gene (4-hydroxy-tetrahydropicolinate reductase) and the *glr* gene (glutamate racemase). In *cagA*- and *cag*PAI-negative strains, this region is called the dg region, and is classified as pre-type A or pre-type B, based on the order of the genes inside the dg region. Using the SouthAfrica7 strain, which has a pre-type B dg region, our analysis showed that SLK40 and SLK91 possess pre-type A dg regions, while SLK36 and SLK260 possess pre-type B (Supplementary Figure S8). This classification is related to the evolution of *cag*PAI and possibly contributes to the level of virulence.

### 3.6. Other Virulence Determinants: Screening for Plasmids, Phages, and Secretion Systems

The plasticity region was analyzed by BLAST against the reference sequences that represent the main type of *tfs*, and then visualized by CGview. These regions were also indicated by the lower GC content (Supplementary Figure S9). We used the plasticity region of the reference strains for genes as follows: P12 for *tfs4a*, Shi470 for *tfs4b*, Gambia94/24 and India7 for *tfs3*, and 26695 for *cag*PAI and *comB*. The complete *cag*PAI was only present in two strains, SLK231 and SLK237, and the *tfs3* and *tfs4* regions were not found in either of these *cag*PAI-positive strains. Incomplete *tfs4a*, *tfs4b*, and *tfs3* regions were observed in SLK36, SLK91, and SLK260. Strain SLK40 did not contain *tfs3*, *tfs4*, or *cag*PAI. The *comB* apparatus for DNA uptake and natural transformation was detected in all strains (Table 2).

No well-known plasmids were identified in any of the Sri Lankan genomes using the PlasmidFinder database. For the phage analysis, a well-known phage was found in only one strain, SLK237, with a GC percentage of 36.1%. However, four strains—SLK231, SLK260, SLK36, and SLK91—showed incomplete types, suggesting the possibility of phages similar to those found in other bacteria (Supplementary Table S2). No phages were detected in the SLK40 strain, in concordance with the absence of *tfs3*, *tfs4*, and *cag*PAI. These three regions have been proposed to play important roles in the transfer of genetic material.

### 3.7. Adherence Ability of Sri Lankan Strains

**Figure 4:**
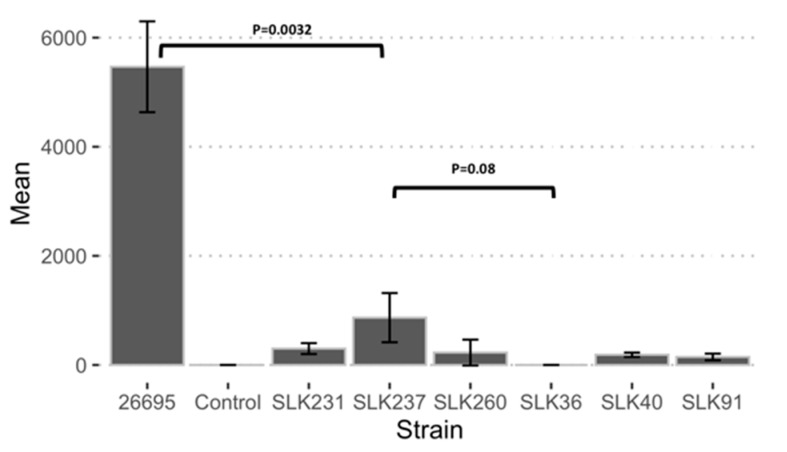
Overview of the presence of *H. pylori* virulence factors in the genome. The *x*-axis shows the strain names and the *y*-axis represents the mean colony-forming unit (CFU) value of three replicates.

Adherence and invasion are the mechanisms used by *H. pylori* for colonization and are associated with the development of gastric cancer and duodenal ulcers [39]. To determine the adherence and invasion abilities of the strains in gastric mucosal tissue, we carried out an adherence assay on AGS cells. The supernatant culture revealed no bacterial growth in the control group, whereas a high bacterial colony-forming unit (CFU) value was observed for strain 26695. All strains isolated from Sri Lanka showed lower adherence. A pairwise comparison between all the Sri Lankan strains showed no significant differences, except between SLK40 and SLK36 (*p* = 0.04). The highest adherence and invasion abilities were found in strain SLK237; however, these were still significantly lower than those of strain 26695 (*p* = 0.0032) (Figure 4).

### 3.8. Antibiotic Susceptibility Test Results

We used an E-test to test for antibiotic susceptibility to five major *H. pylori* antibiotics (AMX, TCN, CAM, MNZ, and LEV) on six *H. pylori* isolates from Sri Lanka. The antibiotic susceptibility test results of the Sri Lankan strains are shown in Table 3. All strains from Sri Lanka were sensitive to AMX, CAM, and TCN. Only one strain, SLK91, showed sensitivity to all five antibiotics tested. The prevalence of LEV resistance was 16.7% (one in six strains), while the prevalence of MNZ resistance was 83.3% (five out of six strains). We defined double-drug resistance as resistance to two of the antibiotics tested. SLK260 was the only multiple-drug-resistant strain, resistant to both LEV and MNZ.

To further investigate the cause of this antibiotic resistance of Sri Lankan strains, we checked for mutations in well-known genes related to these antibiotics. As expected, none of the strains showed any well-known mutations in the 23S rRNA, 16S rRNA, and *pbp1a* genes. The target protein for the quinolone drug levofloxacin is the DNA Gyrase (*gyrA* and *gyrB)*, which is important for the DNA replication. For LEV resistance, we investigated the *gyrA* and *gyrB* genes to search for mutation. An Asn87Lys mutation was observed in the *gyrA* gene of strain SLK260, which could explain the resistance of this strain to LEV.

Metronidazole targets the nitroreductase enzyme and the loss of function of this gene is associated with resistance. However, mutations in other genes, such as *frxA*, *fur*, *omp11*, and *ribF*, have also been reported to be associated with resistance. To understand the high prevalence of resistance to metronidazole, we evaluated five genes—*frxA*, *fur*, *omp11*, *rdxA*, and *ribF*—and aligned these with the ATCC 26,695 control strain. We observed SNPs that were present in the resistant strains and were not observed in the MNZ-sensitive strains. The *rdxA* gene was investigated for any mutational changes or premature termination (Table 4). RdxA is the receptor for MNZ, and any changes to the protein by mutations or variation could affect the drug affinity. An early-stop codon was found at position 52 in SLK231, causing premature termination. The remaining strains had an intact *rdxA* but synonymous point mutations were observed. Alteration of histidine (H) to tyrosine (Y) or threonine (T) at locus 97 was found in 80% (four out of five) of the resistant strains, and this may play a role in metronidazole resistance. Meanwhile, the genes encoding Fur and Omp11 are the most conserved of the five genes evaluated, and the mutations to these genes that were detected are listed in Table 4. Mutations to RibF were also observed, such as Q78R, Q111K, and Q242K.

## 4. Discussion

*H. pylori* is one of the most diverse bacteria, as shown by its high recombination and mutation rates [42,43]. Persistent *H. pylori* infection, leading to co-evolution with humans, has contributed to the wide variety of strains, which show regional characteristics [3]. In this study, a comparative genomics analysis was performed on the genomes of *H. pylori* strains isolated from indigenous people of Sri Lanka. This population is unique in showing a much lower prevalence of *H. pylori* than the worldwide rate of 50%. In Sri Lanka, the prevalence of infection is only 1.7%, and the severity of gastric inflammation is low [18]. However, there had been no reports on the genomic features and virulence factors of *H. pylori* isolates from Sri Lanka. Thus, this study is the first to provide data on the antibiotic resistance, virulence factors, and disease severity of Sri Lankan *H. pylori* strains.

Infection by the strains isolated from Sri Lanka resulted in non-severe inflammation in the hosts, as indicated by histological analysis of the gastric mucosa. Genomic analysis also showed the absence of *cag*PAI as the predominant virulence factor in *H. pylori*, and a less virulent *vacA* genotype (the s2 m2 type). Moreover, the genes encoding BabA and its paralogues were also incomplete. This finding is concordant with a report showing that strains isolated from the South Asian region (Bangladesh and India) had lower pathogenicity than worldwide *H. pylori*, indicating that the genetic composition of the *H. pylori* population affects the virulence properties [44]. Furthermore, the genomes of the Sri Lankan strains were divided into two groups: one close to NAB47 (hpEurope) and one close to SNT49 (hpAsia2) [34,39,40,45]. These results indicate that Sri Lankan strains have similar characteristics to strains isolated from the South Asian region [43,46], suggesting that the *H. pylori* strains found in Sri Lanka may be less of a health concern than those infecting East Asian countries.

CagA and VacA are the major factors of *H. pylori* virulence. The delivery of CagA is dependent on the secretion system encoded by *cag*PAI [47]. A previous study revealed that *cagA* evolution is the result of horizontal gene transfer [48], which was then incorporated into the *H. pylori* genome in the dg region as a cluster of genes, termed *cag*PAI, encoding the secretion apparatus. In the genomes of Sri Lankan strains, there were no traces of the *cag*PAI components in the *cagA*-negative strains. The gene arrangement of the dg region of SLK, classed as pre-type B, is similar to that of strains from hpAfrica1. This pre-type B region can then become occupied by *cag*PAI type B genes, as in hpEurope strains, likely including multiple *cagA* genes [48]. As shown by evaluation of the phylogenetic tree, *cagA* from Sri Lankan strains clustered with *cagA* of hpAsia2 strains, close to hpEurope. This phylogenetic tree supports the suggestion that *cagA* evolved in concordance with the whole genome [49].

In terms of the adherence genes, some Sri Lankan strains did not possess *bab*-paralogous genes, such as *babA*, *babB*, and *babC*. Contrary to this study, when we performed targeted PCR for *bab*-paralogous genes in our previous study, we detected the presence of *babA* in 87% of strains isolated from different regions of South Asia [41]. BabA is a Lewis b binding adhesin required for the colonization of epithelial cells [50,51]. The location of *babA* in locus A is also related to a higher severity of inflammation [41]. The absence of *babA* may contribute to low adherence to AGS cells. In agreement with this, the strains in this study that possessed *babA* showed higher adherence, and thus higher gastric inflammation and atrophy scores.

Alongside a lack of *cagA*, 67% (four out of six) of Sri Lankan strains also showed a less virulent type of *vacA* (s2 i2 m2). There are several genotype markers for the s, i, and m regions, encoding the signal sequence, p33 domain, and p55 domain, respectively [52]. The sequence of the s2 genotype contains an insertion that inhibits vacuolization, and the m2 genotype reduces the specificity of VacA to the cellular receptor. VacA creates vacuoles, detaching the tight junction, and is required for colonization [53]. This may explain the lower severity of the gastritis, as revealed by histology score evaluation, for most of the strains in this study. Unlike *cagA*, the diversity of *vacA* matches the geographic region (Asian versus non-Asian *vacA*), in concordance with our previous study [52,54]. These results support the findings of our previous study, that virulence genes are fixed in indigenous people, including *cagA* and *vacA* [44]. Hence, it is not surprising that the Sri Lankan population maintains the *vacA* s2 m2 genotype in the *cagA*-negative strains.

A variety of disease outcomes are also observed among *cagA*-positive strains, based on the genotype for the EPIYA motifs, which affect the affinity to SHP phosphatase [12]. In this study, both *cagA*-positive Sri Lankan strains possessed a western-type CagA, with one strain having a BCCC-type EPIYA motif. Both EPIYA-A and EPIYA-B have recognition sites for SHP2, and so CagA function may be retained despite an absence of EPIYA-A [12,48]. The EPIYA motifs and the CM motif are binding sites for both SHP2, which induces abnormal cell proliferation, and PAR1, which destroys the gastric mucosa [55]. AGS cells showed a higher proportion of the hummingbird phenotype, with strains with BCCC-type EPIYA-motifs compared with the ABC type. Thus, even though most strains share similar virulence genes, CagA and its EPIYA motifs play integral roles in the virulence of *H. pylori* [56,57].

This study also upholds the consensus that the region of plasticity is a factor in virulence and is associated with more severe gastric inflammation, duodenal ulcers, and gastric cancer [15,16]. Strains with *cag*PAI did not possess other *tfs* genes, while the *cag*PAI-negative strains possessed *tfs* genes that were incomplete or absent. These genes were acquired by horizontal gene transfer, indicated by the negative skew of the GC percentage [47]. The *tfs3* gene produces a protein called CtkA that can be injected into epithelial cells [58]. Moreover, *tfs3* also assists in the uptake of external genetic material [59]. The lack of these *tfs* components is concordant with the absence of plasmids, phages, and IS elements in all strains except SLK237. SLK237 contained IS605 alongside the *tnpA* and *tnpB* genes, which play roles in genomic rearrangement and recombination [60,61].

The antibiotic susceptibility test is key, as it is the most effective way to determine the best regimen of therapy to combat *H. pylori* infection. To our knowledge, this is the first study evaluating the susceptibility of *H. pylori* strains to five major antibiotics in Sri Lanka. We found that all six strains were sensitive to AMX, TCN, and CAM. Of the six strains, five had developed resistance to MNZ, suggesting that a regimen containing MNZ might not be suitable as first-line therapy. The high prevalence of MNZ resistance was not surprising, as resistance to this antibiotic has been reported in many countries worldwide [29,30]. The results of this study suggest that triple therapy containing AMX and CAM is still an effective first-line therapy against *H. pylori* infection in Sri Lanka.

Our results reveal a concerning trend of increasing resistance, particularly towards metronidazole (MNZ) and levofloxacin (LEV). We identified potential mechanisms for this resistance. We detected mutations in the *rdxA* gene, which plays a critical role in the action of MNZ [17]. One strain had a premature-stop codon mutation in this gene, rendering it nonfunctional. Additionally, most resistant strains harbored putative mutations at locus 97, potentially changing histidine to tyrosine or threonine. These mutations might affect the protein structure and hinder MNZ binding [62]. In the case of LEV resistance, a mutation in the *gyrA* gene in locus 87 was identified only in the resistant strains [17,63,64].

The high prevalence of antibiotic resistance in Sri Lankan *H. pylori* strains is a worrying development. Antibiotic overuse is potentially contributing to this emergence of resistance [65,66]. This aligns with observations in other South Asian countries, where similar increases in *H. pylori* resistance have been reported [64]. For example, in Bangladesh, the prevalence of infection is 60.2%, and the resistance to metronidazole, clarithromycin, and levofloxacin is high: 94.6%, 39.3%, and 66.1%, respectively. Nepal also reported high resistance to metronidazole and clarithromycin: 88.1% and 21.4%, respectively. This finding suggests caution should be taken in the use of metronidazole in the *H. pylori* treatment regimens [64]. To combat this growing threat, stricter antibiotic control programs are essential. Promoting public awareness about responsible antibiotic use and exploring alternative treatment options are crucial steps. Further research is needed to understand the specific factors driving resistance in Sri Lanka and to develop effective regional strategies to manage *H. pylori* infections.

This study is limited by the reliability of high-throughput next-generation sequencing. There was also a discrepancy in the number of rRNA and tRNA genes, and a higher number of contigs. This draft genome used short reads to construct a de novo assembly, which might miss some overlapping regions, and the detection of repeated regions or genes with high copy numbers may be problematic [67]. Nevertheless, short-read sequencing provides higher precision and a lower error rate [68,69]. It is also sufficient to comprehensively evaluate the virulome, and this accuracy is necessary for the evaluation of the genotype and gene polymorphisms. Furthermore, the small number of strains evaluated in this study limits the capacity to make causality conclusions; additional research with a larger number of strains is required.

## 5. Conclusions

This investigation, using a comparative genomic method, provides a greater perspective on *H. pylori* virulence factors. Our comparative genomic analysis reveals genes specific to the geographic location, showing less virulent genes in most of the Sri Lankan strains, in concordance with in vitro experiments and histological assessment of gastric inflammation. This study provides a comprehensive insight into the virulence and antibiotic resistance of *H. pylori*. Future research using larger and more diverse population and in vitro experiments on host–pathogen interaction is necessary to establish causality.

## Figures and Tables

**Table 1:** Virulence factors of *H. pylori* isolates from Sri Lanka.

Gene	SLK36	SLK40	SLK91	SLK231	SLK237	SLK260
*cagA*	Absent	Absent	Absent	Present	Present	Absent
EPIYA motif				BCCC	ABC	
CagA type				western	western	
*vacA*	Present	Present	Present	Present	Present	Present
Signal (s)	s2	s2	s2	s1a	s1a	s2
Intermediate (i)	i2	i2	i2	i1	i1	i2
Deletion (d)	d2	d2	d2	d1	d1	d2
Middle (m)	m2	m2	m2	m1a	m1a	m2
C-region (c)	c2	c2	c2	c1	c1	c2
*oipA*	Present	Present	Present	Present	Present	Present
CT repeat	8	7	10	6	6	7
On/off status	On	Off	On	On	On	Off
*iceA1*	Present	Absent	Present	Present	Present	Absent

**Table 2:** Type IV secretion system, *comB* apparatus, and IS elements.

Strain	*tfs4a*	*tfs4b*	*tfs3*	*cagPAI*	*comB*	IS
SLK36	P12	None	None	None	26695	IS21
SLK40	None	None	None	None	26695	IS21
SLK91	None	Shi470	None	None	26695	None
SLK231	None	None	None	26695	26695	IS21, IS605
SLK237	None	None	None	26695	26695	IS21
SLK260	P12	None	Gambia24	None	26695	IS21, IS200/605

**Table 3:** Antibiotic susceptibility test results.

Strain Name	AMX		TCN		CAM		MNZ		LEV	
	MIC	Susc	MIC	Susc	MIC	Susc	MIC	Susc	MIC	Susc
SLK36	0.023	S	<0.016	S	0.023	S	>256	R	<0.02	S
SLK40	0.032	S	0.032	S	0.064	S	192	R	0.094	S
SLK91	0.023	S	0.032	S	0.023	S	0.75	S	0.19	S
SLK231	<0.016	S	0.094	S	0.125	S	48	R	0.5	S
SLK237	0.023	S	0.032	S	0.047	S	24	R	0.19	S
SLK260	0.19	S	0.094	S	0.125	S	>256	R	>32	R

AMX, amoxicillin; TCN, tetracycline; CAM, clarithromycin; MNZ, metronidazole; LEV, levofloxacin; MIC, minimum inhibitory concentration in mg/L; S, sensitive; Susc, susceptibility; R, resistant.

**Table 4:** Mutations in genes related to metronidazole resistance (each row represents one locus that is mutated in one or more strains).

Gene	SLK36	SLK40	SLK91	SLK231	SLK237	SLK260
*frxA*	Premature-stop codon	Premature-stop codon	Truncated locus 1–96	-	Truncated locus 1–96	-
	-	N124S	-	N124S	N124S	-
*fur*	-	-	-	P114H	-	
	-	-	-	-	-	C150Y
*omp11*	-	T13A	-	-	-	-
*rdxA*	-	-	-	Premature-stop codon locus 52	-	-
	R16C	-	-	-	R16C	-
	-	K64N	-	K64N	K64N	-
	H97Y	H97T	-	H97T	H97T	-
	-	P106S	-	P106S	P106S	-
	-	-	-	V204I	V204I	V204I
	-	-	-	D205A	D205A	D205A
*ribF*	-	Q78R	-	Q78R	Q78R	-
	D86E	-	-	-	-	D86E
	-	H94N	-	-	H94N	-
	E109G	-	-	-	-	E109G
	-	Q111K	-	Q111K	Q111K	-
	Q242K	-	-	Q242K	-	Q242K

## Data Availability

Genomes of all strains were deposited in BioProject with the accession number PRJDB17566 and the BioSamples SAMD00753457_SLK231, SAMD00753458_SLK237, SAMD00753459_SLK260, SAMD00753460_SLK36, SAMD00753461_SLK40, and SAMD00753462_SLK91.

## Supplementary Materials

The following supporting information can be downloaded at: https://www.mdpi.com/article/10.3390/microorganisms13020420/s1, Supplementary Figure S1. Evaluation of gastric inflammation in antrum, angulus, and body; Supplementary Figure S2. Visualization of the core genome and shell genome of Sri Lanka strains; Supplementary Figure S3. Presence and absence of virulence genes; Supplementary Figure S4. Determination of *babA*, *babB*, *and babC* locus; Supplementary Figure S5. The phylogenetic tree of *vacA* compared to the *vacA* from the reference genomes; Supplementary Figure S6. Sequence alignment of *cagA* 26695 in the EPIYA region and CM motif; Supplementary Figure S7. The hummingbird phenotype of AGS cells infected with Sri Lanka strains; Supplementary Figure S8. *cag*PAI empty sites arrangement; Supplementary Figure S9. Tfs visualization by CGView; Supplementary Table S1. Quality of assembly by DFAST; Supplementary Table S2. The presence of phage among Sri Lanka isolates.

## References

[1] Mera R.M., Bravo L.E., Camargo M.C., Bravo J.C., Delgado A.G., Romero-Gallo J., Yepez M.C., Realpe J.L., Schneider B.G., Morgan D.R. (2018). Dynamics of *Helicobacter pylori* infection as a determinant of progression of gastric precancerous lesions: 16-year follow-up of an eradication trial. Gut.

[2] Correa P., Houghton J. (2007). Carcinogenesis of *Helicobacter pylori*. Gastroenterology.

[3] Kodaman N., Pazos A., Schneider B.G., Piazuelo M.B., Mera R., Sobota R.S., Sicinschi L.A., Shaffer C.L., Romero-Gallo J., de Sablet T. (2014). Human and *Helicobacter pylori* coevolution shapes the risk of gastric disease. Proc. Natl. Acad. Sci. USA.

[4] Correa P., Piazuelo M.B. (2012). Evolutionary History of the *Helicobacter pylori* Genome: Implications for Gastric Carcinogenesis. Gut Liver.

[5] de Sablet T., Piazuelo M.B., Shaffer C.L., Schneider B.G., Asim M., Chaturvedi R., Bravo L.E., Sicinschi L.A., Delgado A.G., Mera R.M. (2011). Phylogeographic origin of *Helicobacter pylori* is a determinant of gastric cancer risk. Gut.

[6] Hooi J.K.Y., Lai W.Y., Ng W.K., Suen M.M.Y., Underwood F.E., Tanyingoh D., Malfertheiner P., Graham D.Y., Wong V.W.S., Wu J.C.Y. (2017). Global Prevalence of *Helicobacter pylori* Infection: Systematic Review and Meta-Analysis. Gastroenterology.

[7] Fauzia K.A., Tuan V.P. (2024). Rising resistance: Antibiotic choices for *Helicobacter pylori* infection. Lancet Gastroenterol. Hepatol..

[8] Shanks A.M., El-Omar E.M. (2009). *Helicobacter pylori* infection, host genetics and gastric cancer. J. Dig. Dis..

[9] Dai J., Zhao J., Mao L., Hu Y., Lv B. (2022). Study on the value of antibiotic-resistant gene detection in *Helicobacter pylori* in China. Exp. Ther. Med..

[10] Balloux F., Brønstad Brynildsrud O., van Dorp L., Shaw L.P., Chen H., Harris K.A., Wang H., Eldholm V. (2018). From Theory to Practice: Translating Whole-Genome Sequencing (WGS) into the Clinic. Trends Microbiol..

[11] Cross A.S. (2008). What is a virulence factor?. Crit. Care.

[12] Hatakeyama M., Higashi H. (2005). *Helicobacter pylori* CagA: A new paradigm for bacterial carcinogenesis. Cancer Sci..

[13] Ansari S., Yamaoka Y. (2017). Survival of *Helicobacter pylori* in gastric acidic territory. Helicobacter.

[14] Yamaoka Y., Ojo O., Fujimoto S., Odenbreit S., Haas R., Gutierrez O., El-Zimaity H.M., Reddy R., Arnqvist A., Graham D.Y. (2006). *Helicobacter pylori* outer membrane proteins and gastroduodenal disease. Gut.

[15] Backert S., Tegtmeyer N., Fischer W. (2015). Composition, structure and function of the *Helicobacter pylori* cag pathogenicity island encoded type IV secretion system. Future Microbiol..

[16] Backert S., Haas R., Gerhard M., Naumann M. (2017). The *Helicobacter pylori* Type IV Secretion System Encoded by the cag Pathogenicity Island: Architecture, Function, and Signaling. Curr. Top. Microbiol. Immunol..

[17] Tshibangu-Kabamba E., Yamaoka Y. (2021). *Helicobacter pylori* infection and antibiotic resistance—From biology to clinical implications. Nat. Rev. Gastroenterol. Hepatol..

[18] Doohan D., Fauzia K.A., Rathnayake J., Lamawansa M.D., Waskito L.A., Tuan V.P., Dashdorj A., Kabamba E.T., Phuc B.H., Ansari S. (2021). Pepsinogen and Serum IgG Detection Is a Valuable Diagnostic Method for *Helicobacter pylori* Infection in a Low-Prevalence Country: A Report from Sri Lanka. Diagnostics.

[19] Stolte M., Meining A. (2001). The updated Sydney system: Classification and grading of gastritis as the basis of diagnosis and treatment. Can. J. Gastroenterol..

[20] Dixon M.F., Genta R.M., Yardley J.H., Correa P. (1996). Classification and grading of gastritis. The updated Sydney System. International Workshop on the Histopathology of Gastritis, Houston 1994. Am. J. Surg. Pathol..

[21] Uchida T., Kanada R., Tsukamoto Y., Hijiya N., Matsuura K., Yano S., Yokoyama S., Kishida T., Kodama M., Murakami K. (2007). Immunohistochemical diagnosis of the cagA-gene genotype of *Helicobacter pylori* with anti-East Asian CagA-specific antibody. Cancer Sci..

[22] Wick R.R., Judd L.M., Gorrie C.L., Holt K.E. (2017). Unicycler: Resolving bacterial genome assemblies from short and long sequencing reads. PLoS Comput. Biol..

[23] Tanizawa Y., Fujisawa T., Nakamura Y. (2018). DFAST: A flexible prokaryotic genome annotation pipeline for faster genome publication. Bioinformatics.

[24] Gurevich A., Saveliev V., Vyahhi N., Tesler G. (2013). QUAST: Quality assessment tool for genome assemblies. Bioinformatics.

[25] Chen L., Zheng D., Liu B., Yang J., Jin Q. (2016). VFDB 2016: Hierarchical and refined dataset for big data analysis—10 years on. Nucleic Acids Res..

[26] Sayers S., Li L., Ong E., Deng S., Fu G., Lin Y., Yang B., Zhang S., Fa Z., Zhao B. (2019). Victors: A web-based knowledge base of virulence factors in human and animal pathogens. Nucleic Acids Res..

[27] Kumar S., Nei M., Dudley J., Tamura K. (2008). MEGA: A biologist-centric software for evolutionary analysis of DNA and protein sequences. Brief. Bioinform..

[28] Carattoli A., Hasman H. (2020). PlasmidFinder and in silico pMLST: Identification and typing of plasmid replicons in whole-genome sequencing (WGS). Horizontal Gene Transfer.

[29] Arndt D., Grant J.R., Marcu A., Sajed T., Pon A., Liang Y., Wishart D.S. (2016). PHASTER: A better, faster version of the PHAST phage search tool. Nucleic Acids Res..

[30] Siguier P., Pérochon J., Lestrade L., Mahillon J., Chandler M. (2006). ISfinder: The reference centre for bacterial insertion sequences. Nucleic Acids Res..

[31] Stothard P., Wishart D.S. (2005). Circular genome visualization and exploration using CGView. Bioinformatics.

[32] Page A.J., Cummins C.A., Hunt M., Wong V.K., Reuter S., Holden M.T., Fookes M., Falush D., Keane J.A., Parkhill J. (2015). Roary: Rapid large-scale prokaryote pan genome analysis. Bioinformatics.

[33] Hadfield J., Croucher N.J., Goater R.J., Abudahab K., Aanensen D.M., Harris S.R. (2018). Phandango: An interactive viewer for bacterial population genomics. Bioinformatics.

[34] Yahara K., Furuta Y., Oshima K., Yoshida M., Azuma T., Hattori M., Uchiyama I., Kobayashi I. (2013). Chromosome painting in silico in a bacterial species reveals fine population structure. Mol. Biol. Evol..

[35] Petersen A.M., Blom J., Andersen L.P., Krogfelt K.A. (2000). Role of strain type, AGS cells and fetal calf serum in *Helicobacter pylori* adhesion and invasion assays. FEMS Immunol. Med. Microbiol..

[36] Chen Y.-H., Tsai W.-H., Wu H.-Y., Chen C.-Y., Yeh W.-L., Chen Y.-H., Hsu H.-Y., Chen W.-W., Chen Y.-W., Chang W.-W. (2019). Probiotic Lactobacillus spp. act against *Helicobacter pylori*-induced inflammation. J. Clin. Med..

[37] Chang C.-C., Kuo W.-S., Chen Y.-C., Perng C.-L., Lin H.-J., Ou Y.-H. (2016). Fragmentation of CagA reduces hummingbird phenotype induction by helicobactor pylori. PLoS ONE.

[38] The European Committee on Antimicrobial Susceptibility Testing (2015). European Committee on Antimicrobial Susceptibility Testing, Breakpoint Tables for Interpretation of MICs and Zone Diameters.

[39] Kumar N., Mukhopadhyay A.K., Patra R., De R., Baddam R., Shaik S., Alam J., Tiruvayipati S., Ahmed N. (2012). Next-Generation Sequencing and De Novo Assembly, Genome Organization, and Comparative Genomic Analyses of the Genomes of Two *Helicobacter pylori* Isolates from Duodenal Ulcer Patients in India. J. Bacteriol..

[40] Kauser F., Hussain M.A., Ahmed I., Ahmad N., Habeeb A., Khan A.A., Ahmed N. (2005). Comparing genomes of *Helicobacter pylori* strains from the high-altitude desert of Ladakh, India. J. Clin. Microbiol..

[41] Ansari S., Kabamba E.T., Shrestha P.K., Aftab H., Myint T., Tshering L., Sharma R.P., Ni N., Aye T.T., Subsomwong P. (2017). *Helicobacter pylori* bab characterization in clinical isolates from Bhutan, Myanmar, Nepal and Bangladesh. PLoS ONE.

[42] Suerbaum S., Smith J.M., Bapumia K., Morelli G., Smith N.H., Kunstmann E., Dyrek I., Achtman M. (1998). Free recombination within *Helicobacter pylori*. Proc. Natl. Acad. Sci. USA.

[43] Falush D., Kraft C., Taylor N.S., Correa P., Fox J.G., Achtman M., Suerbaum S. (2001). Recombination and mutation during long-term gastric colonization by *Helicobacter pylori*: Estimates of clock rates, recombination size, and minimal age. Proc. Natl. Acad. Sci. USA.

[44] Muñoz-Ramirez Z.Y., Pascoe B., Mendez-Tenorio A., Mourkas E., Sandoval-Motta S., Perez-Perez G., Morgan D.R., Dominguez R.L., Ortiz-Princz D., Cavazza M.E. (2021). A 500-year tale of co-evolution, adaptation, and virulence: *Helicobacter pylori* in the Americas. ISME J..

[45] Kumar N., Mariappan V., Baddam R., Lankapalli A.K., Shaik S., Goh K.-L., Loke M.F., Perkins T., Benghezal M., Hasnain S.E. (2014). Comparative genomic analysis of *Helicobacter pylori* from Malaysia identifies three distinct lineages suggestive of differential evolution. Nucleic Acids Res..

[46] Falush D., Wirth T., Linz B., Pritchard J.K., Stephens M., Kidd M., Blaser M.J., Graham D.Y., Vacher S., Perez-Perez G.I. (2003). Traces of Human Migrations in *Helicobacter pylori* Populations. Science.

[47] Tegtmeyer N., Wessler S., Backert S. (2011). Role of the cag-pathogenicity island encoded type IV secretion system in *Helicobacter pylori* pathogenesis. FEBS J..

[48] Furuta Y., Yahara K., Hatakeyama M., Kobayashi I. (2011). Evolution of cagA oncogene of *Helicobacter pylori* through recombination. PLoS ONE.

[49] Muñoz-Ramírez Z.Y., Mendez-Tenorio A., Kato I., Bravo M.M., Rizzato C., Thorell K., Torres R., Aviles-Jimenez F., Camorlinga M., Canzian F. (2017). Whole Genome Sequence and Phylogenetic Analysis Show *Helicobacter pylori* Strains from Latin America Have Followed a Unique Evolution Pathway. Front. Cell. Infect. Microbiol..

[50] Petzold K., Olofsson A., Arnqvist A., Boren T., Gröbner G., Schleucher J. (2009). *Helicobacter pylori*: How is Adhesin BabA, a Blood Group Antigen Binding Membrane Protein, Involved in Bacterial Adherence?. Biophys. J..

[51] Quintana-Hayashi M.P., Rocha R., Padra M., Thorell A., Jin C., Karlsson N.G., Roxo-Rosa M., Oleastro M., Lindén S.K. (2018). BabA-mediated adherence of pediatric ulcerogenic H. pylori strains to gastric mucins at neutral and acidic pH. Virulence.

[52] Gangwer K.A., Shaffer C.L., Suerbaum S., Lacy D.B., Cover T.L., Bordenstein S.R. (2010). Molecular evolution of the *Helicobacter pylori* vacuolating toxin gene vacA. J. Bacteriol..

[53] Palframan S.L., Kwok T., Gabriel K. (2012). Vacuolating cytotoxin A (VacA), a key toxin for *Helicobacter pylori* pathogenesis. Front. Cell. Infect. Microbiol..

[54] Gutiérrez-Escobar A.J., Bravo M.M., Acevedo O., Backert S. (2019). Molecular evolution of the VacA p55 binding domain of *Helicobacter pylori* in mestizos from a high gastric cancer region of Colombia. PeerJ.

[55] Hatakeyama M. (2017). Structure and function of *Helicobacter pylori* CagA, the first-identified bacterial protein involved in human cancer. Proc. Jpn. Academy. Ser. B Phys. Biol. Sci..

[56] Matos J.I., de Sousa H.A., Marcos-Pinto R., Dinis-Ribeiro M. (2013). *Helicobacter pylori* CagA and VacA genotypes and gastric phenotype: A meta-analysis. Eur. J. Gastroenterol. Hepatol..

[57] Park J.Y., Forman D., Waskito L.A., Yamaoka Y., Crabtree J.E. (2018). Epidemiology of *Helicobacter pylori* and CagA-positive infections and global variations in gastric cancer. Toxins.

[58] Alandiyjany M.N., Croxall N.J., Grove J.I., Delahay R.M. (2017). A role for the tfs3 ICE-encoded type IV secretion system in pro-inflammatory signalling by the *Helicobacter pylori* Ser/Thr kinase, CtkA. PLoS ONE.

[59] Fischer W., Tegtmeyer N., Stingl K., Backert S. (2020). Four chromosomal type IV secretion systems in *Helicobacter pylori*: Composition, structure and function. Front. Microbiol..

[60] He S., Corneloup A., Guynet C., Lavatine L., Caumont-Sarcos A., Siguier P., Marty B., Dyda F., Chandler M., Ton Hoang B. (2015). The IS 200/IS 605 Family and “Peel and Paste” Single-Strand Transposition Mechanism. Microbiol. Spectr..

[61] Abadi A.T.B., Mobarez A.M., Bonten M.J., Wagenaar J.A., Kusters J.G. (2014). Clinical relevance of the cagA, tnpA and tnpB genes in *Helicobacter pylori*. BMC Gastroenterol..

[62] Jenks P.J., Labigne A., Ferrero R.L. (1999). Exposure to Metronidazole In Vivo Readily Induces Resistance in *Helicobacter pylori* and Reduces the Efficacy of Eradication Therapy in Mice. Antimicrob. Agents Chemother..

[63] Fauzia K.A., Aftab H., Tshibangu-Kabamba E., Alfaray R.I., Saruuljavkhlan B., Cimuanga-Mukanya A., Matsumoto T., Subsomwong P., Akada J., Miftahussurur M. (2023). Mutations Related to Antibiotics Resistance in *Helicobacter pylori* Clinical Isolates from Bangladesh. Antibiotics.

[64] Miftahussurur M., Aftab H., Shrestha P.K., Sharma R.P., Subsomwong P., Waskito L.A., Doohan D., Fauzia K.A., Yamaoka Y. (2019). Effective therapeutic regimens in two South Asian countries with high resistance to major *Helicobacter pylori* antibiotics. Antimicrob. Resist. Infect. Control.

[65] Tillekeratne L.G., Bodinayake C.K., Dabrera T., Nagahawatte A., Arachchi W.K., Sooriyaarachchi A., Stewart K., Watt M., Østbye T., Woods C.W. (2017). Antibiotic overuse for acute respiratory tract infections in Sri Lanka: A qualitative study of outpatients and their physicians. BMC Fam. Pract..

[66] Gunasekera Y.D., Kinnison T., Kottawatta S.A., Silva-Fletcher A., Kalupahana R.S. (2022). Misconceptions of antibiotics as a potential explanation for their misuse. A survey of the general public in a rural and urban community in Sri Lanka. Antibiotics.

[67] Alkan C., Sajjadian S., Eichler E.E. (2011). Limitations of next-generation genome sequence assembly. Nat. Methods.

[68] Adewale B.A. (2020). Will long-read sequencing technologies replace short-read sequencing technologies in the next 10 years?. Afr. J. Lab. Med..

[69] Pearman W.S., Freed N.E., Silander O.K. (2020). Testing the advantages and disadvantages of short- and long- read eukaryotic metagenomics using simulated reads. BMC Bioinform..

