# Supplementary material for: Beyond Low Prevalence: Exploring Antibiotic Resistance and Virulence Profiles in Sri Lankan Helicobacter pylori with Comparative Genomics
Source: Microorganisms. 2025 Feb 14;13(2):420. doi: 10.3390/microorganisms13020420 (PMC11858055; doi:10.3390/microorganisms13020420)
Supplement: Supplementary file 1 [file microorganisms-13-00420-s001.zip › microorganisms-3454217-supplementary.pdf]

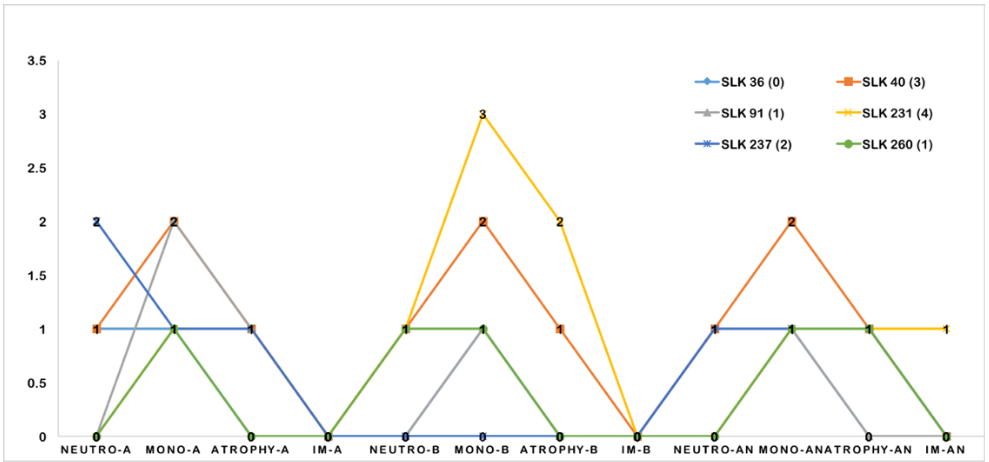

Supplementary Figure S1. Evaluation of gastric inflammation in antrum, angulus, and body. The grades of the neutrophil infiltration, monocyte infiltration, atrophy and intestinal metaplasia were marked as y-axis and each strain is represented by a different color. Overlapping grades was not shown but the strains with the highest value can be observed clearly. Strain`s color is mentioned in the legend and the OLGA score is in the round bracket.

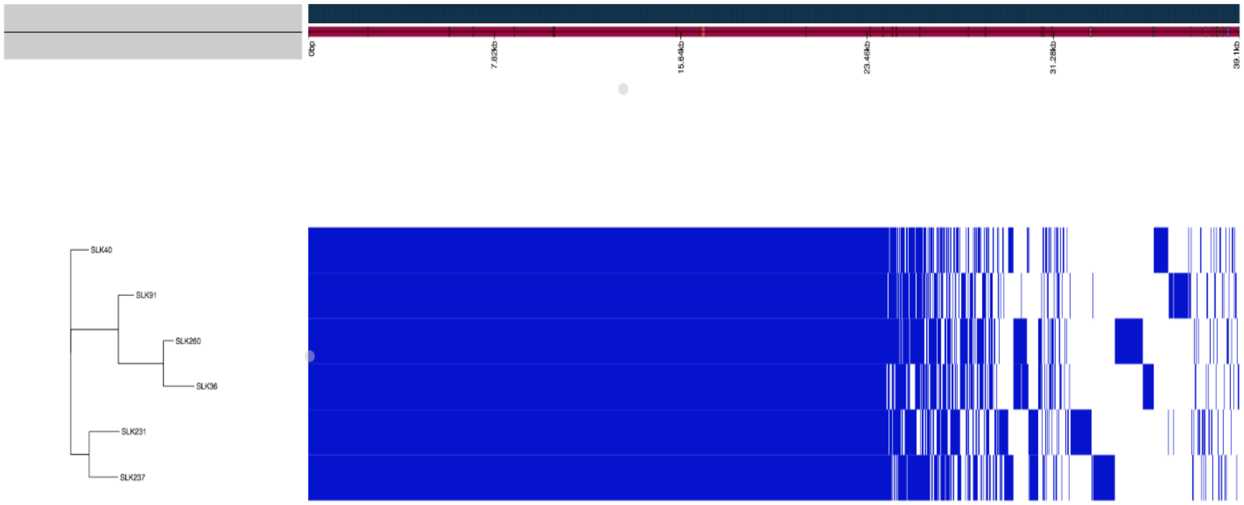

Supplementary Figure S2. Visualization of the core genome and shell genome of Sri Lanka strains. These results showed the Sri Lanka genomes and 26695

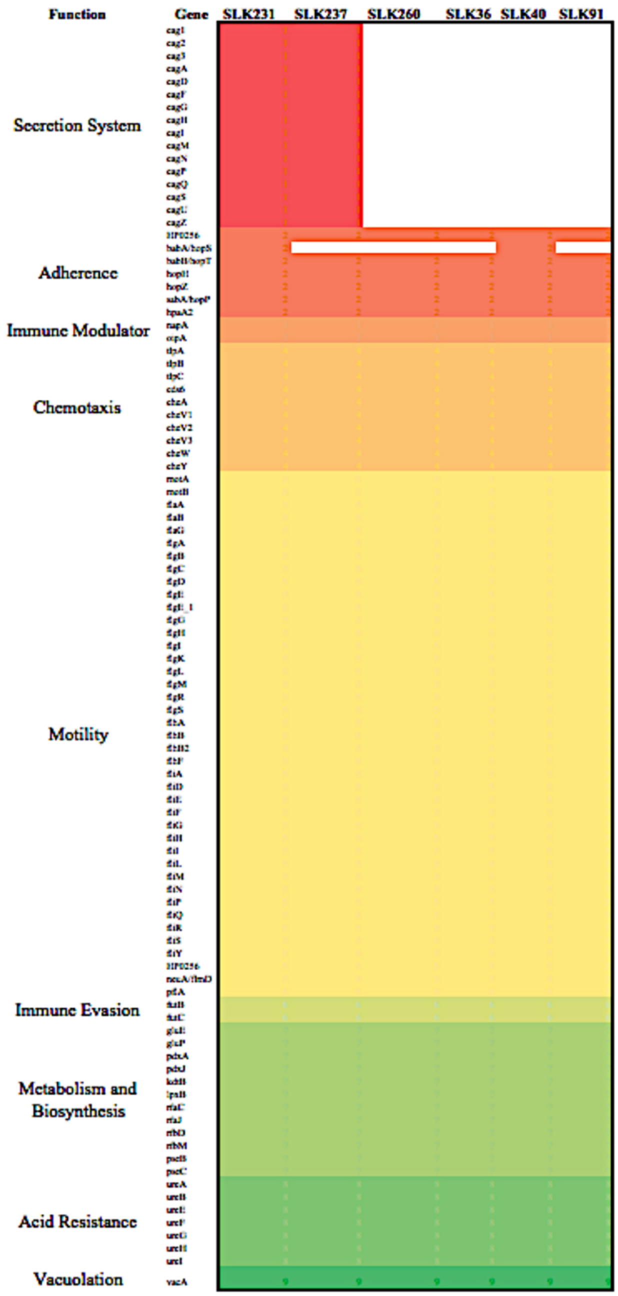

Supplementary Figure S3. Presence and absence of virulence genes. The presence of virulence genes is marked with the color while the absences are represented by the white color. Different colors were used to separate each virulence group according to VFDB.

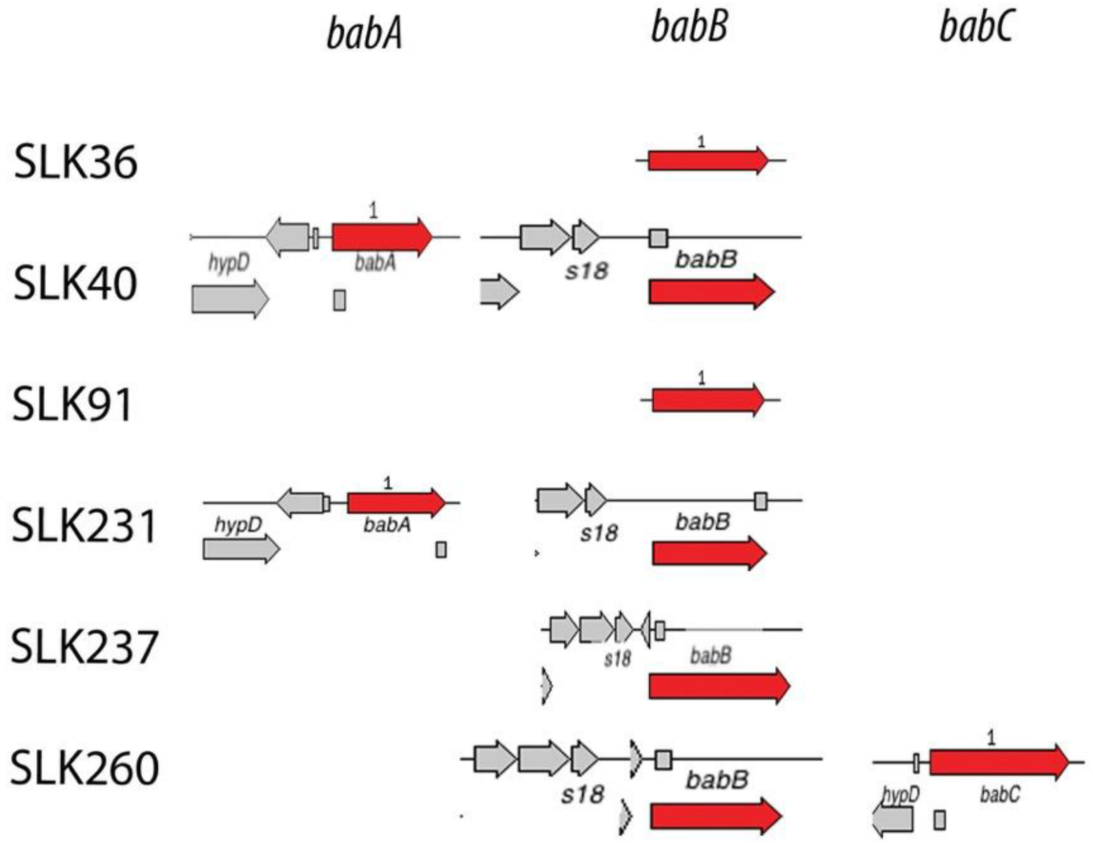

Supplementary Figure S4. Determination of *babA, babB, and babC* locus. This figure showed the position of *bab* gene relative to other genes to determine locus. *bab* gene is located in locus A if located downstram to *hypD* , locus B if the gene is located downstream to *s18*.

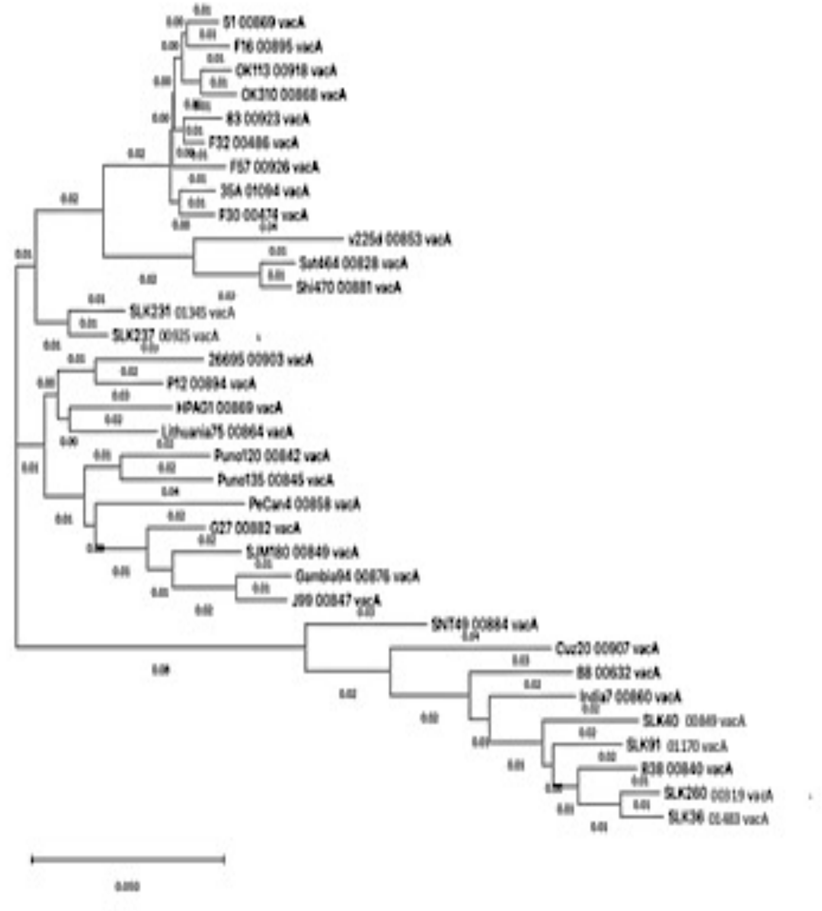

Supplementary Figure S5. The phylogenetic tree of *vacA* compared to the *vacA* from the reference genomes. Sri Lanka strains possessing s1m1 genotype represented in a distinct branch.

cagA Alignment

```
26695  KEQLAQQAQKNEDFNTGKNSELYQSVKNSVNKTLVGNGLSGIEATALAKNFSDIKKELNEKFK
SLK237 KEQLAQQAQKNESFNVGKKSEIYQSVKNGVNGTLVGNGLSKAEATTLSKNFSDIKKELNAKLG
SLK231 KEQLVQQAQKNESFNVGKKSEIYQSVKNGVNGTLVGNGLSKAEATTLSKNFSDIRKELSEKLG

26695  NFNNNN-NGLKNSTEPIYAKVNKKKTGQVASPEEPIYTQVAKKVNAKIDRLNQIASGLGGVGQ
SLK237 NFNNNNNNGLKN--EPIYAKVNKKKAGQAASLEEPIYAQVAKKVNAKIDRLNQIASGLGGVGQ
SLK231 NFNNNN-NGLKN---------------------EPIYAQVAKKVNAKIDRLNQIARGLGGVGQ

26695  AAGFPLKRHD-----------------------------------------------------
SLK237 AAGFPLKRHD----------------------------------------------------
SLK231 AAGFPLKKHDKVDDLSKVGLSASPEPIYATIDDLGGPFPLKKHDKVDDLSKVGLSASPEPIYA

26695  ---------------KVDDLSKVGLSASPEPIYATIDDLGGPFPLKRHDKVDDLSKVGRSRNQ
SLK237 ---------------KVDDLSKVGRSVSPEPIYATIDDLGGPFPLKRHDKVDDLSKVGLSRNR
SLK231 TIDDLGGPFPLKRHDKVDDLSKVGLSASPEPIYATIDDLGGPFPLKRHDKVDDLSKVGLSRNQ
```

Supplementary Figure S6. Sequence alignment of *cagA* 26695 in the EPIYA region and CM motifs

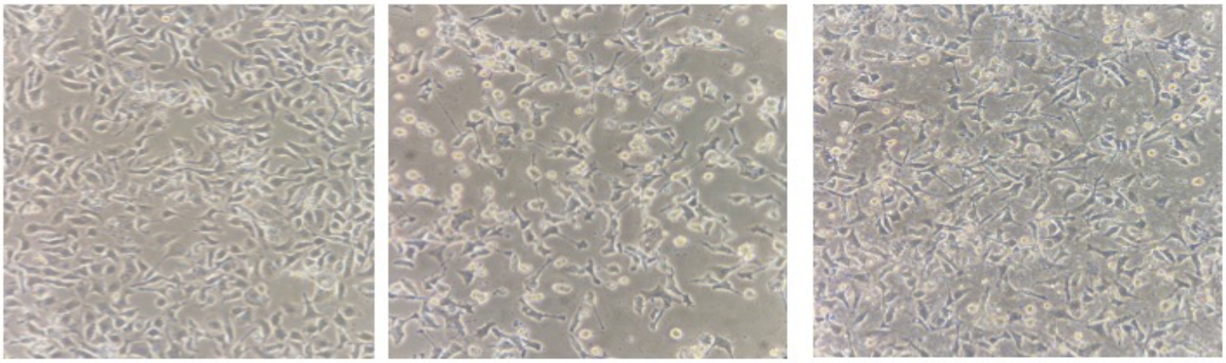

SLK36 SLK231 (BCCC) SLK237 (ABC)

Supplementary Figure S7. The hummingbird phenotype of AGS cells infected with Sri Lanka strains (40X magnification). Each of figure represent the CagA negative, CagA with BCCC genotype and CagA with ABC genotype.

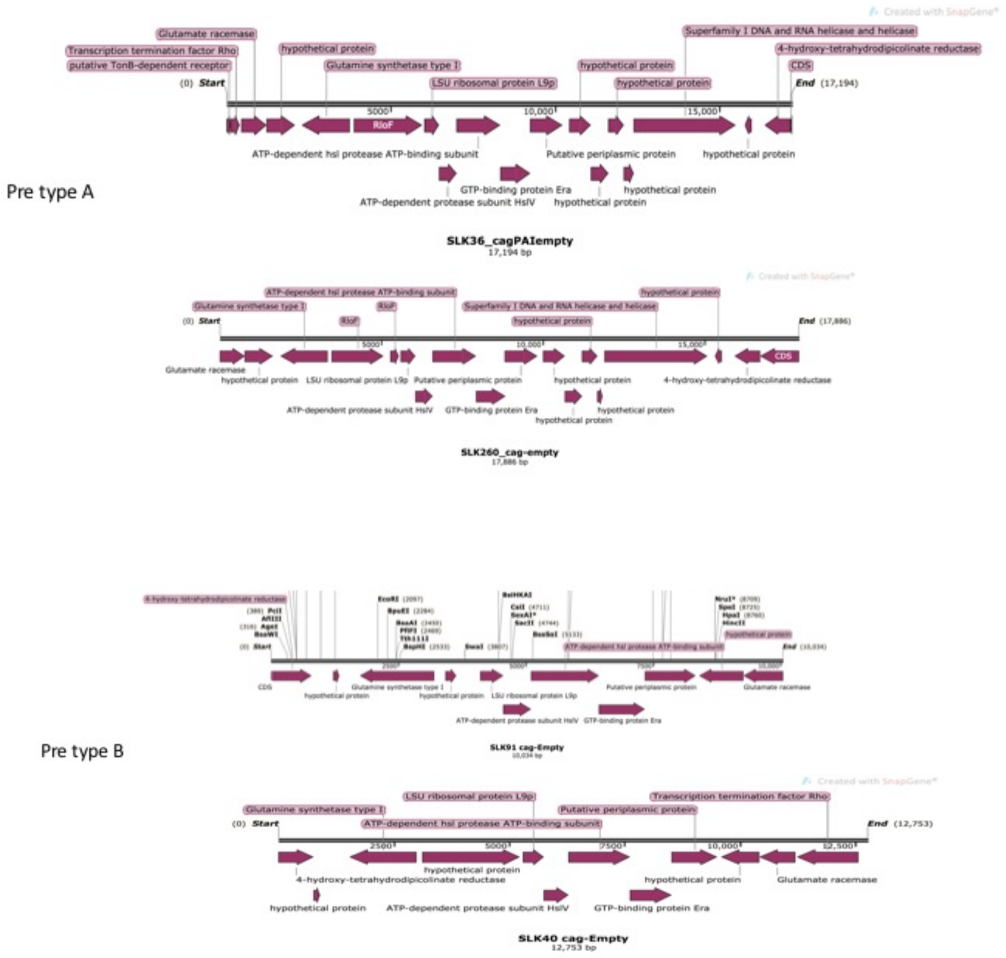

Supplementary Figure S8. *cag*PAI empty sites arrangement

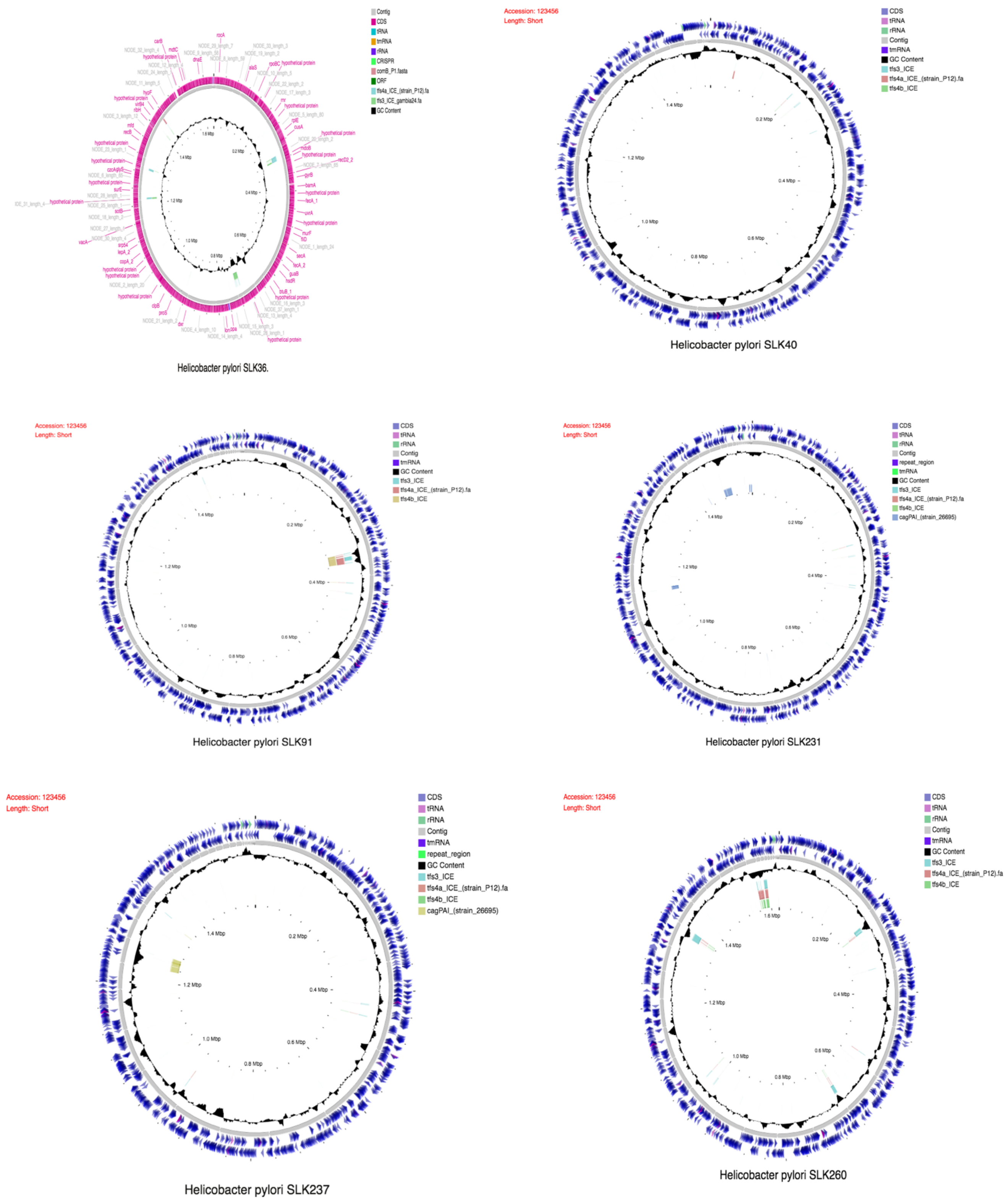

Figure Supplementary S9. The Tfs systems of each strains that are visualized by CGview.

Supplementary Table S1. Quality of assembly by DFAST

	SLK91	SLK40	SLK36	SLK260	SLK237	SLK231	26695
Total Sequence Length (bp)	1552574	1534541	1585718	1625743	1584937	1572153	1667867
Number of Sequences	45	29	48	39	39	48	1
Longest Sequences (bp)	229567	303249	199185	232082	350061	245431	1667867
N50 (bp)	85701	137496	60855	80865	93038	118312	1667867
Gap Ratio (%)	0	0	0	0	0	0	0.00048
GCcontent (%)	39.1	39.2	38.9	38.8	39.1	39.2	38.9
Number of CDSs	1440	1442	1453	1509	1495	1481	1580
Average Protein Length	321.7	321.5	324.3	323.3	318.1	315.7	318.3
Coding Ratio (%)	89.5	90.6	89.1	90	90	89.2	90.5
Number of rRNAs	2	2	2	2	2	2	4
Number of tRNAs	36	36	36	36	36	36	36
Number of CRISPRs	1	0	1	0	1	1	0

Supplementary Table S2. The presence of phage among Sri Lanka isolates

Strains	Phage	Size	Type	GC Percentage	Highest Similarity
SLK36	1	18.9Kb	incomplete	41.55%	PHAGE_Bacill_G_NC_023719(2)
SLK40	None				
SLK91	1	19Kb	incomplete	41.34%	PHAGE_Bacill_AR9_NC_031039[illegible]
SLK231	1	18.9Kb	incomplete	41.55%	PHAGE_Bacill_G_NC_023719(2)
SLK237	1	14.9Kb	intact	36.10%	PHAGE_Helico_KHP30_NC_019[illegible]
	2	18.9Kb	incomplete	41.57%	PHAGE_Bacill_PBS1_NC_04302[illegible]
Slk260	1	19Kb	incomplete	41.35%	PHAGE_Bacill_AR9_NC_031039[illegible]
